# Supplementary material for: Glycolipid recognition and binding by Siglec-6 hinges on interactions with the cell membrane
Source: Commun Biol. 2026 Jan 28;9:333. doi: 10.1038/s42003-026-09609-8 (PMC12953582; doi:10.1038/s42003-026-09609-8)
Supplement: Supplementary file 2 — Supplementary Information [file 42003_2026_9609_MOESM2_ESM.pdf]

Supplementary Material

Glycolipid recognition and binding by Siglec-6 hinges on interactions with the cell membrane

Silvia D'Andrea[1], Edward N. Schmidt[2], Duong Bui[2], Ojas Singh[1], Ling Han[2], Lara K. Mahal[2], John S. Klassen[2], Matthew S. Macauley[2] and Elisa Fadda[3]*

1.Department of Chemistry, Maynooth University, Maynooth, Ireland; 2. Department of Chemistry, University of Alberta, Edmonton, Canada, 3. School of Biological Sciences, University of Southampton, Southampton, United Kingdom.

Confidence level prediction of human Siglec-6 (AF-O43699-F1). To evaluate the reliability of the AlphaFold-predicted Siglec-6 model as a model to start the MD simulations, we analyzed *per*-residue pLDDT confidence values. The AlphaFold model shows high to very high confidence (pLDDT > 70) across the V-set domain (residues 32–144), including the flexible C–C′ loop (residues 70–75) and the KW segment (Lys126, Trp127). The loop and the KW segment display blue and light cyan colors, respectively, supporting the reliability of the geometry used in our analysis, see **Figure S.1.a** and **b.**

Secondary structure analysis of the Siglec-6 V-set domain. We quantified secondary-structure evolution using the DSSP algorithm in AMBER-18 *cpptraj* along the MD trajectories of Siglec-6 bound to GM1 (2.5 μs) and GM2 (2.0 μs, merged replicas). The results indicate that the C–C′ loop, aa 70–75, see **Figure S.1.c**, remains predominantly in a 'coil' conformation, with minor transition to a 'bend' conformation in both complexes. We overlaid representative snapshots from the GM1 (cyan) and GM2 (magenta) simulations, see **Figure S.1.d**. The V-set fold remains stable in both systems, but the C–C′ loop adopts distinct positions relative to the membrane surface, consistent with Trp127 inserting into or withdrawing from the bilayer. These results indicate that changes in loop orientation, not in secondary structure, occur in the complexes with GM1 and GM2.

* For correspondence: Elisa Fadda, elisa.fadda@soton.ac.uk

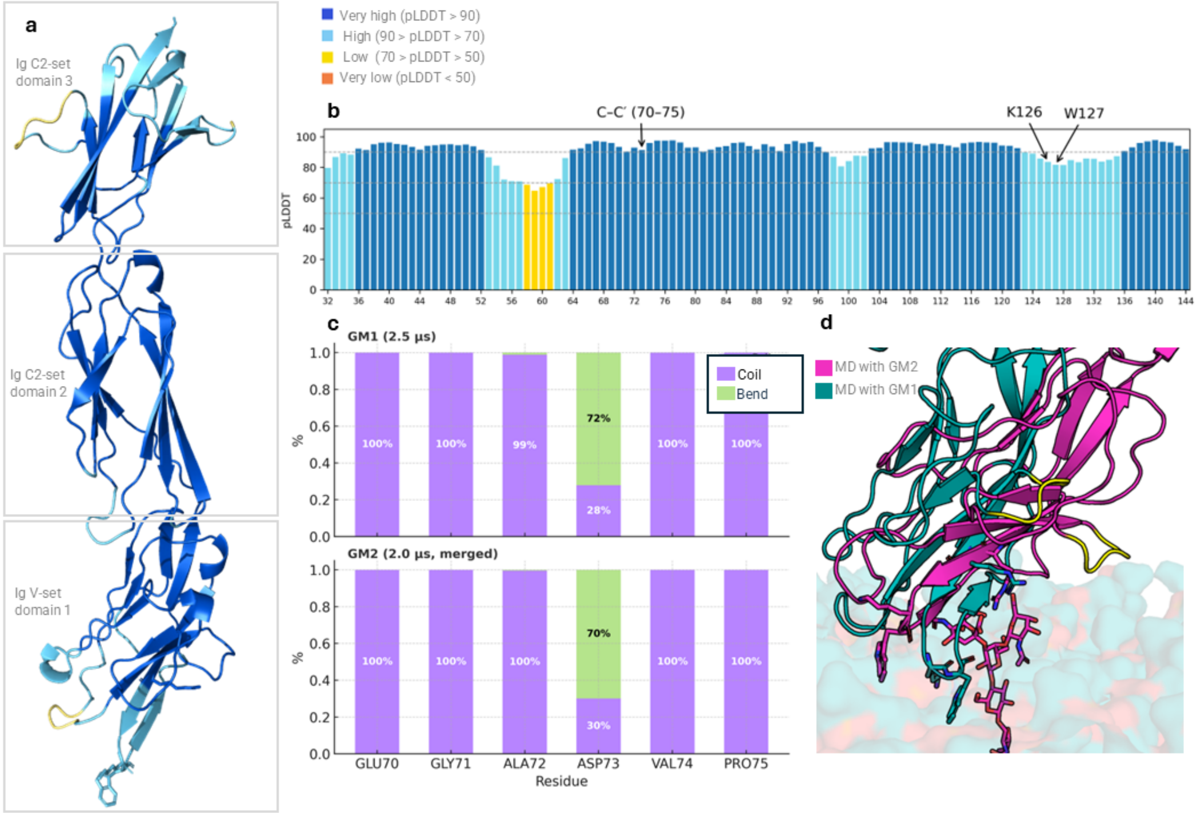

Figure S.1. a. AlphaFold-predicted structure of the Siglec-6 , composed of one Ig V-set and two Ig C2-set domains, colored by pLDDT (B-factor), pLLDT < 50 very low (orange), 50–70 low (yellow), 70–90 high (cyan), > 90 very high (blue). **b.** pLDDT profile of the AlphaFold model aa 32–144 (Ig V set domain), the C–C′ loop (aa 70–75) and the KW segment (Lys126, Trp127) shown by arrows. c) Secondary structure was assigned according to the DSSP algorithm in AMBER-18 *cpptraj* over the MD trajectories of Siglec-6 bound to GM1 (2.5 μs) and GM2 (2.0 μs, merged replicas). The percentage of coil (purple) and bend (green) conformations is reported for each residue. **d**. Structural overlay of representative snapshots from the GM1 system (cyan) and GM2 system (magenta) trajectories showing the conformational stability of the V-set domain on the membrane and the localization and different orientation of the C–C′ highlight in yellow.

MD simulations of the fully *N*-glycosylated Siglec-6 in complex with membrane bound GM1. We ran two independent MD simulations of 1 μs each on a 3D model of the fully glycosylated Siglec-6 in complex with an isolated GM1 ganglioside embedded in the phospholipid bilayer. The molecular composition of the 3D model is described in the Methods section and, aside from the N-glycans, is analogous as the one presented in the main text. The Siglec-6 sequence (453 aa) carries seven *N*-glycosylation sequons, with only one in the V-set domain at Asn103, see **Figure S.2**. All sites are occupied with a biantennary monogalactosylated complex *N*-glycan (GlyTouCan ID G99129GB; 1478.5 Da). As represented in the snapshot from the MD simulations shown in **Figure S.2**, only the *N*-glycans at N104 and N149 interact with the protein, and more specifically engage in contacts that may affect the relative orientation and dynamics of the V-set domain (d1) and the adjacent C2 domain (d2). The RMSD values calculated over the entire extracellular region of

the glycosylated system (i.e. over the backbone atoms of d1, d2, and d3) are higher than the non-glycosylated one, see **Figure S.2.d**. This increase is not due to destabilization by glycans but rather reflects the higher conformational mobility of the distal d3 domain, where glycans such as those at N258 and N295 are not involved in protein contacts and remain solvent exposed and dynamic. It is important to note that d3 is connected to the transmembrane domain, and in a complete cellular context, its dynamics may be constrained by interactions with the membrane. Instead, our data support a stabilizing function of the glycans at N103 and N149, which maintain the relative positioning and rigidity of domains d1 and d2. When compared to the non-glycosylated Siglec-6/GM1 complex, the fully glycosylated Siglec-6 shows a slightly lower average RMSD value relative to the AF (AF-O43699-F1) backbone, suggesting a reduced interdomain flexibility and higher structural stability of the d1–d2 interface, see **Figure S.2.c**. However, the relative flexibility of the system demonstrated by the spread around the median RMSD value in the KDE plots in **Figure S.2.d** for both glycosylated and non-glycosylated systems, precludes defining any clear role of the *N*-glycans in affecting the rigidity of the V-set domain relative to the adjacent C2-domain.

As an important note, the binding to the GM1 is not affected by the *N*-glycosylation neither directly, nor indirectly. This is further supported by the MD simulations of the fully glycosylated Siglec-6/GM1 complex, which consistently show stable anchoring on the membrane through the interactions with Trp127 and Lys126.

Membrane-insertion analyses of Trp127 and Lys126, see **Figure S.2.f.**, show similar depth and persistence in glycosylated and non-glycosylated complexes, confirming that ligand engagement is unchanged; the main glycan effect is to decrease d1–d2 mobility, yielding more ordered, less variable, behavior in the RMSD/KDE results. In replica MD1, Trp127 briefly disengaged from the bilayer after about 1 μs, coinciding with the loss of the Arg–Neu5Ac contact and destabilization of the complex.

In replica MD2, the Arg–Neu5Ac interaction was lost after 700 ns, as in the non-glycosylated run, but the complex remained stable thanks to persistent Trp127/Lys126 anchoring and a recurring contact between the terminal Gal of GM1 and Asp70 in the C–C′ loop, as shown in **Figure S.2.e-f**.

Distance traces (**Figure S.2.e.**) and insertion profiles (**Figure S.2.f.**) indicate that V-set domain glycans do not affect GM1 binding, but they reduce inter-domain flexibility and enhance membrane interaction stability.

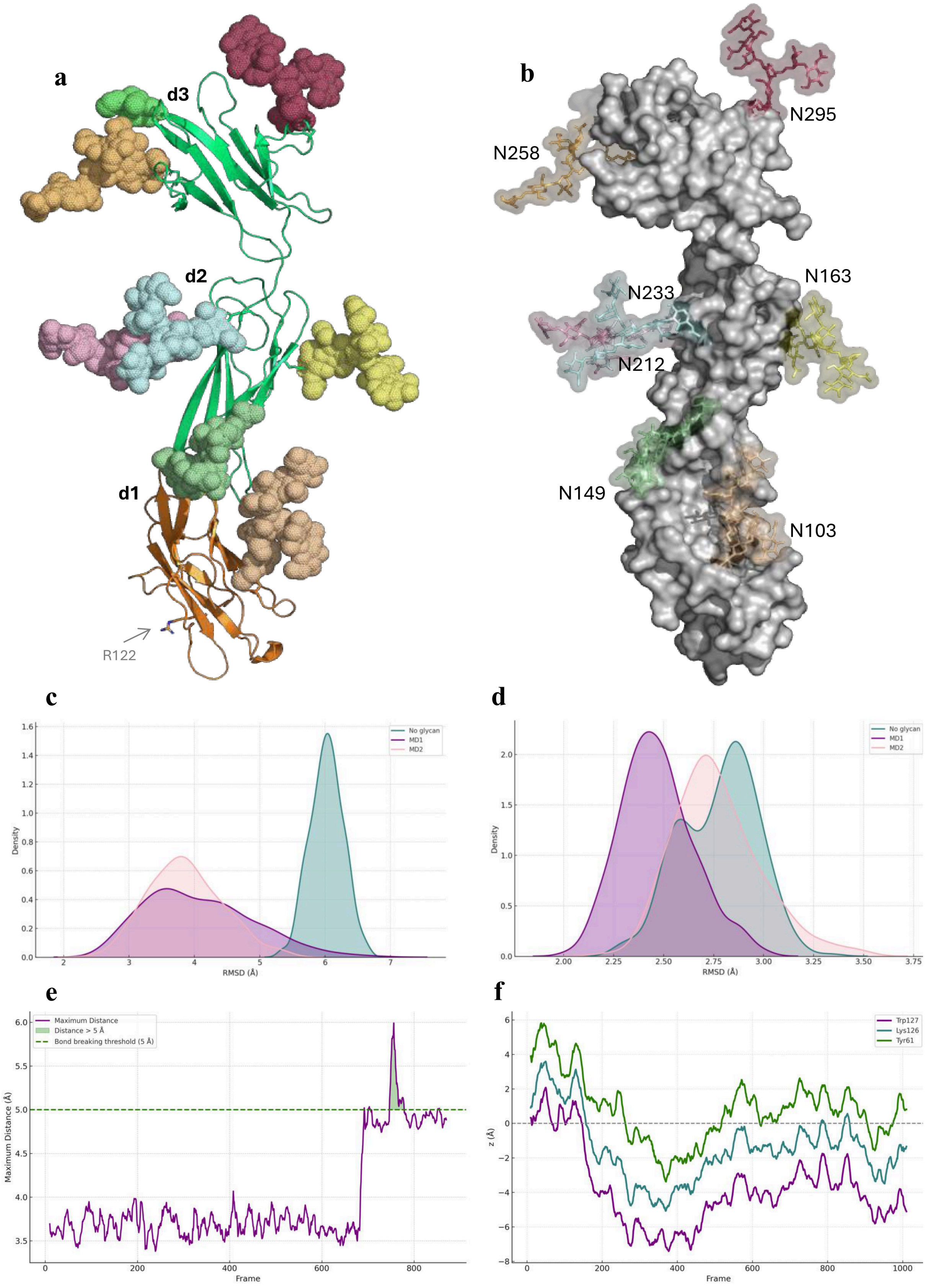

Figure S.2. a. 3D structure of the Siglec-6 extracellular region from AF model AF-O43699-F1, shown as cartoons. The terminal V-set binding domain (d1) is shown in orange and the C2-set domains (d2 and d3) are shown in green. *N*-linked glycans are rendered with atoms as vdW spheres and color-coded individually. The

seven *N*-glycosylation sequons were assessed as occupied with the GlcNAc Scanning tool in GlycoShape ReGlyco[5]. **b.** Representative snapshot of the Siglec-6 from the MD simulation with the N-glycosylation sites labelled. **c.** Kernel density estimates (KDE) distributions of the backbone RMSD values along the trajectory, corresponding to the Siglec-6 calculated for d1 and d2. The results corresponding to the non-glycosylated Siglec-6 are shown in green and for the fully glycosylated Siglec-6 in purple and pink for MD1 and MD2, respectively). **d.** Kernel density estimates (KDE) distributions of the backbone RMSD values calculated along the MD trajectories for the backbone of the Siglec-6 extracellular region, i.e. d1 to d3. **e.** Time evolution along the MD trajectory of the distance (Å) between the Arg122 and the Neu5Ac carboxylate group. Data points correspond to the largest distance value calculated between four pairs of atoms R122-NH1(2) and O11(12)-Neu5Ac. A distance of 5 Å was chosen as a threshold for the formation of the salt bridge. **f.** Membrane-insertion depth (z, Å) of Trp127, Lys126 relative to the DSPC phosphorus plane (z = 0, dashed). Negative values indicate insertion into the bilayer. Molecular rendering with *pymol* (www.pymol.org) and KDE plots with *seaborn* and *Matplotlib* (https://seaborn.pydata.org/).

Assessing the potential involvement of non-canonical Arg residues in the V-set domain of Siglec-6 in the recognition and binding of GM1os. Previous work by some of us[1] clearly showed that Siglec-6 binds gangliosides with a mechanism independent of the canonical Arg, namely Arg122. Due to the presence of multiple Arg and Lys residues in the V-set domain of Siglec-6, it is reasonable to hypothesize that one of those may have replaced or could complement the function of the conserved Arg, as some of us recently demonstrated in the case of Siglec-10[2]. To address this matter, we ran a set of three uncorrelated molecular dynamics (MD) simulations of the non-glycosylated Siglec-6 (see results in the main text) in an equilibrated water box, in the presence of seven GM1os molecules. The starting structure in all replicas was built with one of the GM1os bound to the canonical Arg122 to occupy the canonical site, while another was positioned in proximity to Arg92, , see **Figure S.3,** which has been previously highlighted as a potential (non-canonical) binding Arg[1]. We focused our attention to the Arg in the V-set domain but monitored contacts with all Arg on the Siglec-6 accessible surface, as these could be involved in both *cis,* and *trans* binding events. The results show that none of the GM1os, except the one bonded to the canonical Arg122, engage in stable interactions, see **Figure S.3**. The Arg92 is engaged in a stable salt bridge with Asp115, which is never disrupted during any of the three independent MD simulations. As a caveat, it is important to underline that additive force fields, such as the one we used to run the MD simulations in this work, are notoriously biased towards enhancing electrostatic contacts[3] and thus tend to overestimate the stability of already strong hydrogen bonding interactions, such as salt bridges[4]. In this specific case an analysis of the Siglec-6 3D structure indicates that such salt bridge interaction may be essential to the correct folding of the V-set domain, and mutation of Arg92 (or of Asp115) would compromise the folding, which explains the low expression levels of the R92A mutant reported in earlier work[1].

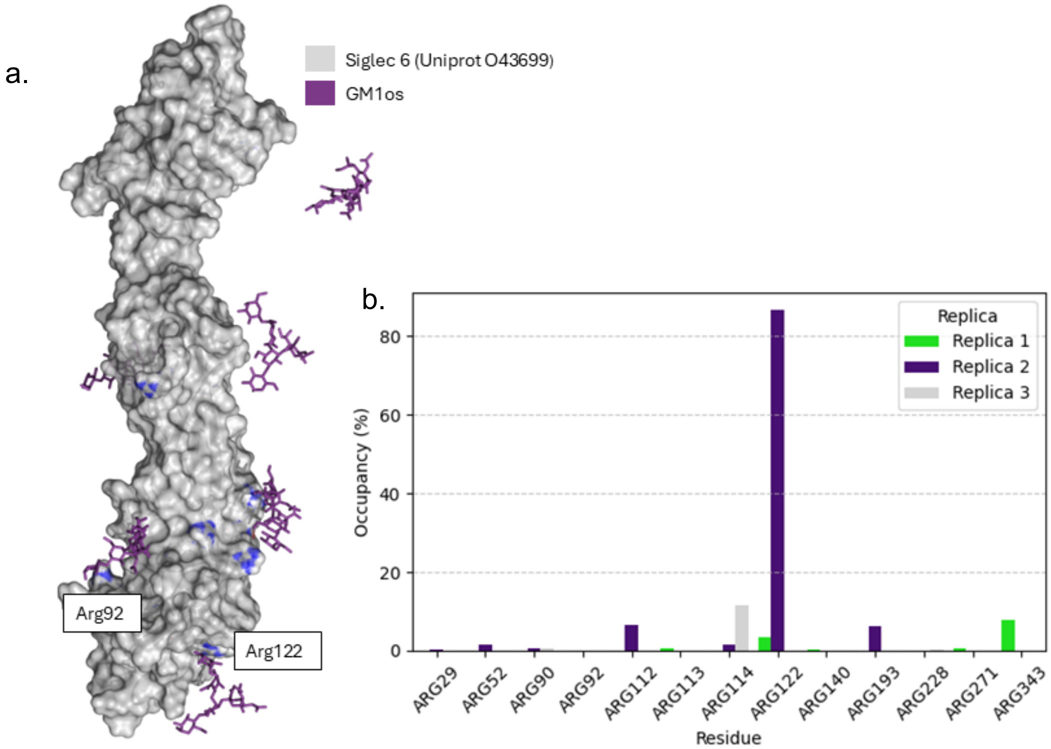

Figure S.3. a: 3D surface representation of Siglec-6 (AF-O43699, gray) with seven GM1os molecules (purple sticks), including one placed at the canonical sialic acid binding site (Arg122) and others distributed in possible non canonical regions of the protein. **b:** Bar plot showing the occupancy of GM1os interactions with selected ARG residues across three independent MD replicas. Occupancy is assessed in terms of distance between guanidinium C (CZ) of Arg and the carboxyl carbon (C1) of the Neu5Ac with a threshold of 5 Å for binding. Molecular rendering with VMD (https://www.ks.uiuc.edu/Research/vmd/) and bar plot with MS Excel.

MATERIALS AND METHODS

Computational Methods

MD simulations of Siglec-6 with multiple GM1os. The 3D structure of Siglec-6 was obtained from the AlphaFold database (AF-O43699-F1) [6,7], and the structures of GM1os were retrieved from the GlycoShape database [5]. One GM1os molecule was manually placed at the canonical sialic acid-binding site near Arg122, while six additional GM1os molecules were randomly distributed around the protein surface to investigate for potential alternative binding sites. The protein was parameterized using the AMBER ff14SB force field, and the glycans with GLYCAM06j-1. Simulations were performed using AMBER-18 . Following 500,000 steps of steepest descent minimization, the system was heated in two stages (0–100 K and 100–300 K, 500 ps each) in the NVT ensemble using Langevin dynamics (γ_ln = 1.0 ps^{-1}). Equilibration was carried out in the NPT ensemble for 500 ps at 1 atm using a Berendsen barostat. All restraints on the protein heavy atoms were then removed, and three independent 300 ns production runs were performed from different initial velocities. Due to intrinsic limitations of the force field in modeling systems with multiple free glycans, the simulations were halted prematurely [3]. Residue specific occupancies were calculated by monitoring persistent contacts (within 5 Å) between the carboxyl group of the sialic acid and the guanidinium group of ARG side chains over time.

MD simulations of isolated GM1 and GM3 in a lipid bilayer. Two independent systems were prepared, each embedding either a GM1 and GM3 ganglioside within a symmetric 130 Å × 130 Å lipid bilayer composed of 60% 1,2-distearoyl-sn-glycero-3-phosphocholine (DSPC) and 40% cholesterol (CHL1), using the CHARMM-GUI Membrane Builder tool[8]. All molecular dynamics (MD) simulations were performed with AMBER18, using the CHARMM36m force field to parameterize the gangliosides and lipids. Each system underwent energy minimization for 5000 steps (2500 steps of steepest descent followed by 2500 steps of conjugate gradient minimization). Equilibration followed the six-step CHARMM-GUI protocol [9], during which positional restraints of 10 kcal/mol·Å² were initially applied and then gradually reduced to 5 kcal/mol·Å² for the protein and 2.5 kcal/mol·Å² for the membrane, before being completely removed. Temperature was maintained at 315.15 K using Langevin dynamics (γ_ln = 1.0 ps^{-1}), and pressure was controlled semi-isotropically at 1 atm using the Berendsen barostat. Periodic boundary conditions were applied throughout. Long-range electrostatics were treated using the Particle Mesh Ewald (PME) method with an 11 Å cutoff. Bond lengths involving hydrogen atoms were constrained using the SHAKE algorithm, enabling a 2 fs integration time step. Each system was simulated for 1 μs. The

orientation of the sialic acid headgroup (Neu5Ac) was quantified by calculating the tilt angle (θ), defined as the angle between the vector connecting atom C1 of the terminal galactose and atoms C2 and C3 of the Neu5Ac residue, relative to the axis perpendicular to the membrane plane. Tilt angle distributions were computed over the entire MD trajectories for both GM1 and GM3.

MD simulations of GM1 and GM2 in complex with Siglec-6. The Siglec-6/GM1 complex was constructed by aligning the conformation of GM1, obtained from the membrane embedded simulation, to the sialic acid moiety in the crystal structure of Siglec-3 bound to a sialoside analogue (PDB: 7AW6). The structure of Siglec-6 (AF-O43699) was validated by structural alignment with Siglec-3, resulting in a backbone RMSD of 0.704 Å. A second system, containing GM2, was generated by removing the terminal Gal residue from GM1. Both complexes were embedded in the same DSPC/cholesterol bilayer described above, and the simulations were carried out using AMBER v.22 [10], following the same multistep equilibration and production protocol. In the GM1 system, a distance restraint (5 kcal/mol·Å²) was applied between the side chain of Arg122 and the carboxyl group of the Neu5Ac to stabilize the salt bridge during the initial 400 ns of the 2.5 μs production simulation. The same approach was used for the GM2 complex; in both 1-μs replicas, removal of the restraint led to destabilization of the complex, indicating a loss of stable interaction between Siglec-6 and GM2 in the absence of the terminal Gal residue. All MD trajectories were analysed using Python3 scripts written in-house. Distances, occupancies, and tilt angles were calculated frame-by-frame using the *cpptraj* module in AMBER v.22 and the graphical user interface VMD[11]. Kernel Density Estimates (KDE) and all other plots were generated using the *matplotlib* (https://matplotlib.org/) and *seaborn* (https://seaborn.pydata.org/) libraries.

Modelling of the glycosylated Siglec-6

The AlphaFold-predicted structure of Siglec-6 (AF-O43699-F1) was scanned using GlcNAc Scan tool[5], which identified seven putative *N*-glycosylation sites across the extracellular region. The glycan 3D structures were retrieved from the GlycoShape database and added to each predicted sequon using Re-Glyco, which optimizes the orientation of the attached N-glycans (GlyTouCan ID: G99129GB) to minimize steric clashes and ensure proper linkage geometry. The glycosylated PDB output from Re-Glyco was then used as input in CHARMM-GUI Membrane Builder to generate the membrane system. MD simulations were performed

as described in the Methods section “MD simulations of GM1 and GM2 in complex with Siglec-6”.

Experimental Methods

Nanodisc preparation

Nanodiscs (NDs), consisting of 10% GM1 and DMPC, were prepared using the protocol described by Sligar and coworkers [12]. Briefly, the lipids were diluted in methanol at the desired molar ratios, dried under gentle vacuum to form a lipid film and then resuspended in a buffer (pH 7.4) containing 20 mM TrisHCl, 0.5 mM EDTA, 100 mM NaCl and 25 mM sodium cholate (Sigma-Aldrich Canada, Oakville, Canada). The membrane scaffold protein MSP1E1 was added to the mixture at a MSP1E1:(GM1+DMPC) molar ratio of 1:100. The ND self-assembly process was initiated by adding pre-washed biobeads (Bio-Rad, Mississauga, Canada) and the mixture was incubating at room temperature overnight on an orbital shaker. After incubation, the supernatant was recovered and the NDs purified using a Superdex 200 10/300 size-exclusion column (GE-Healthcare Life Sciences, Piscataway, NJ) equilibrated with 200 mM ammonium acetate (pH 7.4). Finally, the ND fraction was collected, concentrated and dialyzed into 200 mM ammonium acetate (pH 7.4) using an Amicon microconcentrator (EMD Millipore, Billerica, MA) with a 30 kDa MW cut-off. All ND stock solutions were stored at –80 °C prior to analysis. Each ND sample consists of two copies of MSP1E1 and ~200 lipids. Therefore, ND concentration was estimated based on the UV absorption of MSP1E1 at 280 nm with the extinction coefficient of $\varepsilon_{280nm,MSP1E1}$ = 32,430 $cm^{-1}\cdot M^{-1}$, and assuming [ND] = 1/2×[MSP1E1]. Each ND approximately contained 20 GM1 molecules and 180 DMPC molecules.

Concentration independent native mass spectrometry with catch-and-release

The Concentration Independent native mass spectrometry (COIN-nMS) assay, performed with Catch-and-Release (CaR), was implemented in negative ion mode using a Q-Exactive Ultra-High Mass Range (UHMR) Orbitrap mass spectrometer (Thermo Fisher Scientific, Bremen, Germany) equipped with a modified nanoflow electrospray ionization (nanoESI) source, as described elsewhere[13].

Catch-and-release native mass spectrometry with ion mobility separation

CaR-nMS, performed with Ion Mobility Separation (IMS), was implemented in negative ion mode using a Waters Synapt G2S quadrupole-ion mobility separation-time of flight (Q-IMS-

TOF) mass spectrometer (Waters, Manchester, UK) equipped with a NanoLockSpray ion source. A nanoESI voltage of –0.8 kV, a source temperature of 80 °C and a cone voltage of 70 V were used. Argon was used in the Trap and Transfer ion guides at pressures of 2.77 × 10^{-2} and 2.84 × 10^{-2} mbar, respectively, with the Trap and Transfer voltages of 5 and 2 V, respectively. For IMS, a wave height of 40 V and a wave velocity of 650 m s^{-1} were applied along with a helium and nitrogen (IMS gas) gas flow of 120 and 90 mL min^{-1}, respectively. Collision-induced dissociation (CID) by isolating ions with m/z 7,000±100 in the quadrupole, subjecting them to IMS, and then collisional activation using a 100 V collision energy in the Transfer region [14]. All data was processed using MassLynx software (v4.1) in combination with DriftScope v2.5.

Mutagenesis. Mutagenesis was achieved by three consecutive polymerase chain reactions using gene-overlap extension mutagenesis. The first reaction used a forward primer at the start of the gene containing a 5’ *NheI* site and reverse primer that was centered on the mutation site and the complete gene of interest at the template. The second reaction used a forward prime that was centered on the mutation site and a reverse primer at the end of the gene that featured and 3’ *AgeI* site and used the gene of interest as the template. The third reaction used the products of the first two PCR reactions as a template and the primers at the start and end of the gene. Following size validation using agarose gel electrophoresis, the final PCR products were digested with *NheI* and *AgeI* and then ligated into pCDNA5. Following ligation, the ligation product was then transformed into *E. coli* DH5α. Colonies were then grown in LB overnight at 37 °C and then miniprepped. The minipreps were then validated by restriction digest and Sanger sequencing.

Table S.1: Primers used in this work.

**Primer Name**	**Sequence**
Siglec-6 Fwd	AGC AGC GCT AGC ATG CAG GGA GCC CAG GAA GCC
Siglec-6 Rvs	AGC AGC ACC GGT TCA CTT GTG TAT CTT GAT TTC
Sig-6 K124A Fwd	CGG TTG AAG TCC GCA TGG ATG AAA TAC

Sig-6 K124A Rvs	GTA TTT CAT CCA TGC GGA CTT CAA CCG
Sig-6 Rvs 3D	AGC AGC ACC GGT CCT GCC TTC TGG TTT CCA ATG
Sig-6 K126A Fwd	CGG TTG AAG TCC GCG TGG ATG AAA TAC GG
Sig-6 K126A Rvs	CCG TAT TTC ATC CAC GCG GAC TTC AAC CG
Sig-6 W127A Fwd	GTT GAA GTC CAA AGC CAT GAA ATA CGG TTA TAC
Sig-6 W127A Rvs	GTA TAA CCG TAT TTC ATG GCT TTG GAC TTC AAC
Sig-6 K129A Fwd	GTC CAA ATG GAT GGC ATA CGG TTA TAC
Sig-6 K129A Rvs	GTA TAA CCG TAT GCC ATC CAT TTG GAC

Cell culture. Flp-In Chinese hamster ovary (CHO) cells were cultured as previously described[1], but in short, CHO cells were cultured in DMEM/F12 Media (Gibco) supplemented with 5% (V/V) fetal bovine serum (Gibco), penicillin (100 U/mL), and streptomycin (100 µg/mL). Cells were grown at 37 °C and 5% CO_2 in tissue culture dishes.

Stable transfection of Flp-In CHO Cells. See main manuscript for details.

Liposomes preparation. Liposomes were prepared by hydrating lipid thin consisting of DSPC (61.5 mol%), cholesterol (38 mol%), and PEG_{45}-DSPE (0.4 mol) and AF647-PEG_{45}-DSPE films with PBS pH 7.4. Glycolipids were added at the expense of DSPC. The lipid solutions were extruded using an Avanti-mini extruder using 800 nm and 100 nm filters respectively. Liposomes were then validated with dynamic light scattering. More details regarding liposome production can be found elsewhere[1],[15].

Siglec-Fc expression and purification. See main manuscript for details.

Cell assay. 150,000 cells were added to a 96 well U-bottom microplate. Liposome solutions were prepared at 50 μM in 1% (g:mL) BSA PBS pH 7.4. 50 μL of liposome solution was added to the cells and the cells were incubated with the liposomes for 30 min at 37 °C. The cells were then washed with PBS twice. Following the washes, the cells were then resuspended in anti-Siglec antibody solution (1/250 V:V antibody in 500 μM EDTA, 1% BSA (g:mL)). The cells were then incubated at 4 °C for 30 min. The cells were again washed and then analyzed by flow cytometry. Antibody information can be found in Table S.2, and general gating strategy can be found in Figure S.3.

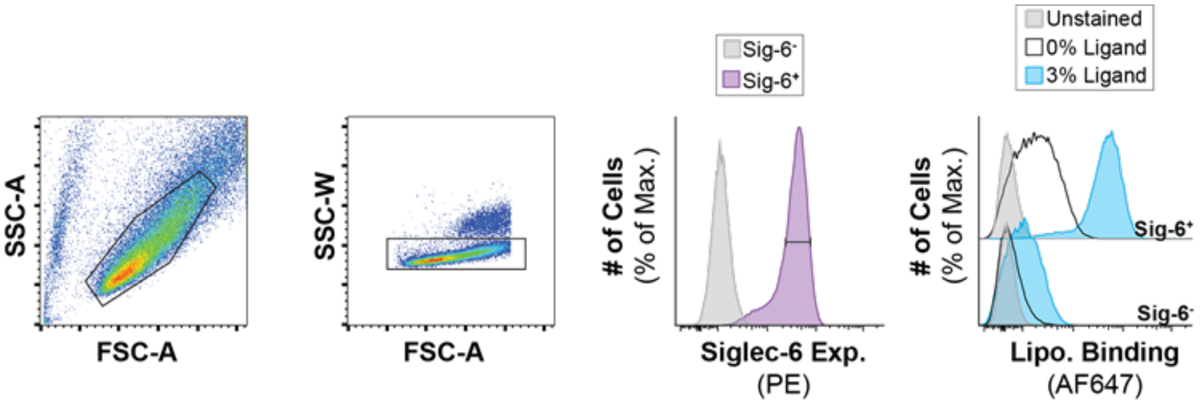

Figure S.3. Gating strategy used to measure glycolipid liposome binding to Chinese hamster ovary cells expressing Siglec-6 mutants. Liposome binding was measured using 3 mol% compound NGL1 [1].

The chemical structure of neoglycolipid ligand (NGL1) used in this experiment and the synthesis are described in the main manuscript and also in ref.[1].

Table S.2: Antibodies used in this work

Antibody	Supplier	Cat. No.	Label	Clone	Isotype	Dilution
anti-Siglec-6	R&D Systems	FAB2859P	PE	767329	Mouse IgG2A	1:250 (V:V)

Flow cytometry. Flow cytometry measurements were collected on a 5-laser Fortessa X-20 (BD Bioscience). All the resulting data were analyzed using FlowJo (10.5.3) software (BD Biosciences)

Siglec-Fc ELISA. A detailed description of the approach can be found elsewhere [1,15]. In short, 50 µL of 50 µM glycolpid solution was transferred to a 96 well ELISA microplate. The plate was left at 37 °C for 2 to remove the ethanol. The wells were blocked with 5% (g:mL) bovin serum albumin (BSA) for 1 h at room temperature. During the blocking step, the Siglec-Fc-Strep-Tactin solution (Siglec-Fc solution: 2 µg/mL, Strep-Tactin-HRP: 0.13 µg/mL-IBA) was prepared and complexed at room temperature for at least 30 min at room temperature. The blocking buffer was then removed, and the Siglec-Fc solution was added for 2 h at room temperature. Unbound Siglec-Fc solution was then removed, and the amount of binding was measured using TMB substrate. The reaction was then quenched using 1 M H_3PO_4 and the Abs_{450} was measured using a Molecular Devices SpectraMAX iD5.

References

(1) Schmidt, E. N.; Lamprinaki, D.; McCord, K. A.; Joe, M.; Sojitra, M.; Waldow, A.; Nguyen, J.; Monyror, J.; Kitova, E. N.; Mozaneh, F.; Guo, X. Y.; Jung, J.; Enterina, J. R.; Daskhan, G. C.; Han, L.; Krysler, A. R.; Cromwell, C. R.; Hubbard, B. P.; West, L. J.; Kulka, M.; Sipione, S.; Klassen, J. S.; Derda, R.; Lowary, T. L.; Mahal, L. K.; Riddell, M. R.; Macauley, M. S. Siglec-6 Mediates the Uptake of Extracellular Vesicles through a Noncanonical Glycolipid Binding Pocket. *Nat. Commun.* **2023**, *14* (1), 2327.

(2) Sobczak, K.; Antoñana-Vildosola, A.; Valverde, P.; Travecedo, M. A.; Jame-Chernaboo, Z.; Schmidt, E. N.; D'Andrea, S.; Valdaliso-Díez, E.; Oyenarte, I.; Laugieri, M. E.; Joe, M.; Mozaneh, F.; Lin, S.-Y.; Bosch, A.; Moure, M. J.; Franconetti, A.; Lee, S. Y.; de Durana, J. E.-D.; Pérez-Gutierrez, L.; Palazón, A.; Marcelo, F.; Fadda, E.; Corzana, F.; Gimeno, A.; Macauley, M. S.; Jiménez-Barbero, J.; Ereño-Orbea, J. The Unique Molecular Recognition Features of Siglec-10: Structural Insights into Sialoglycan and Antibody Interactions. *bioRxiv*, 2025. https://doi.org/10.1101/2025.06.10.658867.

(3) Molecular Simulations of Complex Carbohydrates and Glycoconjugates. *Current Opinion in Chemical Biology* **2022**, *69*, 102175.

(4) Ahmed, M. C.; Papaleo, E.; Lindorff-Larsen, K. How Well Do Force Fields Capture the Strength of Salt Bridges in Proteins? *PeerJ* **2018**, *6*, e4967.

(5) Ives, C. M.; Singh, O.; D'Andrea, S.; Fogarty, C. A.; Harbison, A. M.; Satheesan, A.; Tropea, B.; Fadda, E. Restoring Protein Glycosylation with GlycoShape. *Nat Methods* **2024**, *21* (11), 2117–2127.

(6) Jumper, J.; Evans, R.; Pritzel, A.; Green, T.; Figurnov, M.; Ronneberger, O.; Tunyasuvunakool, K.; Bates, R.; Žídek, A.; Potapenko, A.; Bridgland, A.; Meyer, C.; Kohl, S. A. A.; Ballard, A. J.; Cowie, A.; Romera-Paredes, B.; Nikolov, S.; Jain, R.; Adler, J.; Back, T.; Petersen, S.; Reiman, D.; Clancy, E.; Zielinski, M.; Steinegger, M.; Pacholska, M.; Berghammer, T.; Bodenstein, S.; Silver, D.; Vinyals, O.; Senior, A. W.; Kavukcuoglu, K.; Kohli, P.; Hassabis, D. Highly Accurate Protein Structure Prediction with AlphaFold. *Nature* **2021**, *596* (7873), 583–589.

(7) Varadi, M.; Anyango, S.; Deshpande, M.; Nair, S.; Natassia, C.; Yordanova, G.; Yuan, D.; Stroe, O.; Wood, G.; Laydon, A.; Žídek, A.; Green, T.; Tunyasuvunakool, K.; Petersen, S.; Jumper, J.; Clancy, E.; Green, R.; Vora, A.; Lutfi, M.; Figurnov, M.; Cowie, A.; Hobbs, N.; Kohli, P.; Kleywegt, G.; Birney, E.; Hassabis, D.; Velankar, S. AlphaFold Protein Structure Database: Massively Expanding the Structural Coverage of Protein-Sequence Space with High-Accuracy Models. *Nucleic Acids Res.* **2022**, *50* (D1), D439–D444.

(8) Jo, S.; Kim, T.; Iyer, V. G.; Im, W. CHARMM-GUI: A Web-Based Graphical User Interface for CHARMM. *J Comput Chem* **2008**, *29* (11), 1859–1865.

(9) Lee, J.; Cheng, X.; Swails, J. M.; Yeom, M. S.; Eastman, P. K.; Lemkul, J. A.; Wei, S.; Buckner, J.; Jeong, J. C.; Qi, Y.; Jo, S.; Pande, V. S.; Case, D. A.; Brooks, C. L., 3rd; MacKerell, A. D., Jr; Klauda, J. B.; Im, W. CHARMM-GUI Input Generator for NAMD, GROMACS, AMBER, OpenMM, and CHARMM/OpenMM Simulations Using the CHARMM36 Additive Force Field. *J Chem Theory Comput* **2016**, *12* (1), 405–413.

(10) Case, D. A.; Metin Aktulga, H.; Belfon, K.; Ben-Shalom, I.; Berryman, J. T.; Brozell, S. R.; Cerutti, D. S.; Cheatham, T. E., III; Andrés Cisneros, G.; Cruzeiro, V. W. D.; Darden, T. A.; Duke, R. E.; Giambasu, G.; Gilson, M. K.; Gohlke, H.; Goetz, A. W.; Harris, R.; Izadi, S.; Izmailov, S. A.; Kasavajhala, K.; Kaymak, M. C.; King, E.; Kovalenko, A.; Kurtzman, T.; Lee, T.; LeGrand, S.; Li, P.; Lin, C.; Liu, J.; Luchko, T.; Luo, R.; Machado, M.; Man, V.; Manathunga, M.; Merz, K. M.; Miao, Y.; Mikhailovskii, O.; Monard, G.; Nguyen, H.; O'Hearn, K. A.; Onufriev, A.; Pan, F.; Pantano, S.; Qi, R.; Rahnamoun, A.; Roe, D. R.; Roitberg, A.; Sagui, C.; Schott-Verdugo, S.; Shajan, A.; Shen, J.; Simmerling, C. L.; Skrynnikov, N. R.; Smith, J.; Swails, J.; Walker, R. C.; Wang, J.; Wang, J.; Wei, H.; Wolf, R. M.; Wu, X.; Xiong, Y.; Xue, Y.; York, D. M.; Zhao, S.; Kollman, P. A. *Amber 2022*; University of California, San Francisco, 2022.

(11) Humphrey, W.; Dalke, A.; Schulten, K. VMD: Visual Molecular Dynamics. *J. Mol. Graph.* **1996**, *14* (1), 33–38, 27–28.

(12) Ritchie, T. K.; Grinkova, Y. V.; Bayburt, T. H.; Denisov, I. G.; Zolnerciks, J. K.; Atkins, W. M.; Sligar, S. G. Chapter 11 - Reconstitution of Membrane Proteins in Phospholipid Bilayer Nanodiscs. *Methods Enzymol* **2009**, *464*, 211–231.

(13) Bui, D. T.; Favell, J.; Kitova, E. N.; Li, Z.; McCord, K. A.; Schmidt, E. N.; Mozaneh, F.; Elaish, M.; El-Hawiet, A.; St-Pierre, Y.; Hobman, T. C.; Macauley, M. S.; Mahal, L. K.; Flynn, M. R.; Klassen, J. S. Absolute Affinities from Quantitative Shotgun Glycomics Using Concentration-Independent (COIN) Native Mass Spectrometry. *ACS Cent. Sci.* **2023**, *9* (7), 1374–1387.

(14) Li, J.; Fan, X.; Kitova, E. N.; Zou, C.; Cairo, C. W.; Eugenio, L.; Ng, K. K. S.; Xiong, Z. J.; Privé, G. G.; Klassen, J. S. Screening Glycolipids Against Proteins in Vitro Using Picodiscs and Catch-and-Release Electrospray Ionization-Mass Spectrometry. *Anal Chem* **2016**, *88* (9), 4742–4750.

(15) Schmidt, E. N.; Guo, X. Y.; Bui, D. T.; Jung, J.; Klassen, J. S.; Macauley, M. S. Dissecting the Abilities of Murine Siglecs to Interact with Gangliosides. *J. Biol. Chem.* **2024**, *300* (7), 107482.

(16) Schmidt, E. N.; Jung, J.; Macauley, M. S. Flow Cytometry-Based Detection of Siglec Ligands. *Methods Mol. Biol.* **2023**, *2657*, 181–193.
