# Supplementary material for: Glycolipid recognition and binding by Siglec-6 hinges on interactions with the cell membrane
Source: Commun Biol. 2026 Jan 28;9:333. doi: 10.1038/s42003-026-09609-8 (PMC12953582; doi:10.1038/s42003-026-09609-8)
Supplement: Supplementary file 3 — Description of Additional Supplementary Files [file 42003_2026_9609_MOESM3_ESM.pdf]

Description of Additional Supplementary File

File name: Supplementary Data

Description: Data points corresponding to experiments in Figure 3a,b, Figure 4a,b and Figure 5a
